# Use of Synonymous Deoptimization to Derive Modified Live Attenuated Strains of Foot and Mouth Disease Virus

**DOI:** 10.3389/fmicb.2020.610286

**Published:** 2021-01-21

**Authors:** Fayna Diaz-San Segundo, Gisselle N. Medina, Edward Spinard, Anna Kloc, Elizabeth Ramirez-Medina, Paul Azzinaro, Steffen Mueller, Elizabeth Rieder, Teresa de los Santos

**Affiliations:** ^1^Plum Island Animal Disease Center (PIADC), Agricultural Research Service, United States Department of Agriculture, Greenport, NY, United States; ^2^Kansas State University College of Veterinary Medicine, Manhattan, KS, United States; ^3^PIADC Research Participation Program, Oak Ridge Institute for Science and Education, Oak Ridge, TN, United States; ^4^Department of Pathobiology and Veterinary Science, University of Connecticut, Storrs, CT, United States; ^5^Codagenix, Inc., Farmingdale, NY, United States

**Keywords:** FMDV, synonymous deoptimization, codon bias, vaccine, attenuation, FMD

## Abstract

Foot-and-mouth disease (FMD) is one of the most economically important viral diseases that can affect livestock. In the last 70 years, use of an inactivated whole antigen vaccine has contributed to the eradication of disease from many developed nations. However, recent outbreaks in Europe and Eastern Asia demonstrated that infection can spread as wildfire causing economic and social devastation. Therefore, it is essential to develop new control strategies that could confer early protection and rapidly stop disease spread. Live attenuated vaccines (LAV) are one of the best choices to obtain a strong early and long-lasting protection against viral diseases. In proof of concept studies, we previously demonstrated that “synonymous codon deoptimization” could be applied to the P1 capsid coding region of the viral genome to derive attenuated FMDV serotype A12 strains. Here, we demonstrate that a similar approach can be extended to the highly conserved non-structural P2 and P3 coding regions, providing a backbone for multiple serotype FMDV LAV development. Engineered codon deoptimized P2, P3 or P2, and P3 combined regions were included into the A_24_Cruzeiro infectious clone optimized for vaccine production, resulting in viable progeny that exhibited different degrees of attenuation in cell culture, in mice, and in the natural host (swine). Derived strains were thoroughly characterized *in vitro* and *in vivo*. Our work demonstrates that overall, the entire FMDV genome tolerates codon deoptimization, highlighting the potential of using this technology to derive novel improved LAV candidates.

## Introduction

Foot-and-mouth disease (FMD) is a highly contagious viral disease that affects cloven-hoofed animals. Its etiologic agent is the FMD virus (FMDV), a member of the *Picornaviridae* family (Grubman and Baxt, 2004). The virus genome consists of a single-strand positive sense RNA molecule of approximately 8500 nucleotides in length that encodes for a long open reading frame (ORF) flanked by untranslated regions (UTR) at the 5′ and 3′ ends. The ORF is mainly divided in four regions, the Leader protein, and P1, P2, and P3 immature polyproteins. Processing of the polyproteins by three viral encoded proteinases (Lpro, 2A, and 3Cpro) yield four structural proteins, namely 1A (VP4), 1B (VP2), 1C (VP3), 1D (VP1), and non-structural (NS), Lpro, 2A, 2B, 2C, 3A, 3 distinct copies of 3B (VPg), 3Cpro, and the RNA-dependent RNA polymerase 3Dpol, mature proteins. FMDV can infect over 70 animal species including domestic and wild animals, mostly cattle, swine, sheep, goat, and deer (Grubman and Baxt, 2004). FMD is listed by the International Organization of Animal Health (OIE) as a reportable disease and upon disclosure of an outbreak, severe limitation of movement, and trading are imposed (OIE, 2018). Control of disease in FMD-free countries usually involves culling of infected and in-contact animals, animal movement restrictions, thorough disinfection of affected premises and, sometimes, vaccination with an inactivated virus vaccine (Pluimers et al., 2002). Animals are prophylactically vaccinated in countries where the disease is endemic. Although harmless to human health, FMD outbreaks cause severe economic loss. For example, the 2001 United Kingdom outbreak carried an economic burden that exceeded US$12B and impacted the economy of affected areas and the nation as a whole (Mahy, 2004).

The commercially available vaccine is a preparation of binary ethylenimine (BEI)-inactivated whole virus formulated with adjuvants (Doel, 2003). However, novel recombinant vaccines are under development. Among the most advanced, a replication defective human adenovirus 5 that delivers empty FMDV capsids (Ad5-FMD) has been evaluated in recent years obtaining a conditional license in the United States (Grubman et al., 2010). Similarly to the inactivated vaccine, the Ad5-FMD vaccine requires about 7 days to protect cattle and swine against FMDV infection providing a relatively short-lived immunity as compared to the immunity induced by natural infection (Grubman and Baxt, 2004). The Global Foot-and-Mouth Disease Research Alliance (GFRA), an international organization whose ultimate mission is eradication of FMD, has set the development of next generation improved vaccines and biotherapeutics, as one of their five most important goals. Historically it has been shown that live attenuated vaccines (LAVs) confer rapid and long-lasting protection against viral infection (Minor, 2015). Indeed, LAVs against smallpox and rinderpest viruses have resulted in the eradication of these viruses (Glynn and Glynn, 2004; Roeder et al., 2013; Greenwood, 2014). Similar approaches have been used for measles to reduce virus propagation in some parts of the world (Moss and Strebel, 2011). We and others have explored this possibility by introducing mutations or deletions in different regions of the viral genome that encode virulence factors (Chinsangaram et al., 1998; Uddowla et al., 2012; Kloc et al., 2017; Rai et al., 2017; de los Santos et al., 2018). For instance, substitutions of two conserved amino acid residues in SAF-A/B, Acinus and PIAS (SAP) domain within the NS viral protease L^pro^ coding region of FMDV A12 rendered a highly attenuated mutant virus (de los Santos et al., 2009; Wang et al., 2011). Interestingly, when the modified attenuated strain was evaluated as a LAV candidate in swine, complete protection against challenge with wild type virus was observed even at 2 days post vaccination (Diaz-San Segundo et al., 2012). However, the small number of attenuating mutations in the SAP mutant poses a considerable risk for reversion to virulence. This is not surprising, given the high rate of mutation for RNA viruses; ergo, a larger number of attenuating mutations should be considered in the design of the ideal LAV candidate. More recently, and based on previous studies done for FMDV A_12_ (Mason et al., 1997; Chinsangaram et al., 1998), a FMDV A_24_ vaccine candidate engineered with a deletion of the L^pro^ coding region (leaderless virus, FMDVLL_3__B__3__D_) containing DIVA markers built in the NS proteins 3B and 3D, and with capacity to swapping capsid-coding sequences, has been described (Uddowla et al., 2012). The FMDVLL_3__B__3__D_ virus was incapable of causing FMD in cattle and pigs and failed to spread the disease to contact animals when applied live (Uddowla et al., 2012; Eschbaumer et al., 2020). However, BEI inactivated FMDVLL_3__B__3__D_ vaccine was very effective in cattle and is currently at early stages of development as a safe chemically inactivated vaccine platform (Hardham et al., 2020), but not as a LAV due to its limited replication and therefore low induced immunity when applied as a live virus in the natural host.

A relatively new approach for the construction of LAVs involves the use of synonymous deoptimization. All biological systems are characterized by a determined frequency of unequal synonymous codon usage for each amino acid, resulting in a particular codon bias (Ikemura, 1981). Independently of the “codon bias” concept, synonymous codon-pairs might also be used at frequencies that differ from those statistically predicted, a phenomenon that has been defined as “codon-pair bias” and is species specific (Moura et al., 2007). By applying this technology to recode the nucleotides of polio, influenza, respiratory syncytial and dengue viruses, viable viruses were derived by reverse genetics displaying severe attenuation *in vitro* and *in vivo* (Coleman et al., 2008; Mueller et al., 2010; Yang et al., 2013; Le Nouën et al., 2014; Shen et al., 2015; Stauft et al., 2018, 2019a,b). Furthermore, applying codon-pair bias deoptimization to other viruses such as, porcine respiratory and reproductive syndrome virus (Park et al., 2020), classical swine fever virus (Velazquez-Salinas et al., 2016), human immunodeficiency type 1 virus (Martrus et al., 2013), Zika virus (Li et al., 2018), or vesicular stomatitis virus (Wang et al., 2015), has also resulted in attenuated strains with potential for LAV development. In proof of concept studies, we demonstrated that codon-pair bias deoptimization is tolerated by FMDV (Diaz-San Segundo et al., 2016). Deoptimization of the P1 structural protein coding region resulted in a highly attenuated FMDV strain (A12-P1Deopt) in mice that when administered at relatively low doses induced a protective immune response against lethal challenge with wild type (WT) FMDV, offering a “safety margin” of about 10,000. Virulence studies demonstrated that A12-P1Deopt virus was also attenuated in the natural host. A 100x higher dose of A12-P1Deopt relatively to A12-WT virus, was required to induce similar disease in swine. Additionally, significantly high levels of neutralizing antibodies (Abs) could be detected in sera predicting protection against FMD.

Foot-and-mouth disease virus structural proteins influence significant aspects of virus infection and immunity including those involved in capsid assembly and stability, virus binding to target cells, and antigenic specificity (Grubman and Baxt, 2004). The high level of variability in FMDV capsid proteins, as represented by the multiple serotypes and subtypes of this virus, reflects FMDV exposure to selective pressures and adaptation (Domingo et al., 2003; Carrillo et al., 2005). In contrast, other regions of the viral genome are more conserved and can be substituted between serotypes in infectious FMDV clones without affecting viability (Almeida et al., 1998; Uddowla et al., 2012, 2013; Lee et al., 2017). Manipulation of more conserved regions of the viral genome to generate attenuation would allow synthesis of a viral backbone that could be consistently used to generate chimeras with the P1 structural regions of the different serotypes. In fact, Uddowla et al. (2012) described the use of restriction enzymes flanking the P1 region that could be used for high throughput capsid swapping.

Here, we demonstrate that stable viable attenuated strains of FMDV that grow up to WT endpoint titers in tissue culture can also be produced by deoptimizing more conserved non-structural P2/P3 regions. Furthermore, introduction of restriction sites for easy capsid exchange (Uddowla et al., 2012) to allow for the construction of multiple serotype variants, were well tolerated in the P2/P3 deoptimized strains. Among the different obtained viable mutants, A24-P2/P3Deopt was the most attenuated and induced an adaptive immune response *in vivo*, in mice and swine. These results confirm the use of codon deoptimization to recode FMDV nucleotide sequences as a strategy to reduce its virulence. Importantly, inclusion of capsid swapping capabilities and introduction of DIVA markers, makes this approach an attractive platform for further development into modified live attenuated FMDV vaccine candidates.

## Materials and Methods

### Cells and Viruses

Porcine kidney cell lines (LF-PK and IBRS-2) were obtained from the Foreign Animal Disease Diagnostic Laboratory (FADDL), Animal, Plant, and Health Inspection Service (APHIS) at the PIADC. Porcine kidney cells expressing specific integrins (LF-PKαVβ6) were developed in house by LaRocco et al., 2015. Secondary porcine kidney (PK) or embryonic bovine kidney (EBK) cells were supplied by the APHIS National Veterinary Services Laboratory, Ames, IA, United States. BHK-21 cells (baby hamster kidney cells strain 21, clone 13, ATCC CL10), were purchased from the American Type Culture Collection (ATCC, Rockville, MD, United States). All cells were maintained as previously described (de los Santos et al., 2009). Stock of FMDV A24-WT was generated from the full-length serotype A_24_Cruzeiro infectious clone (pA_24_-WT) and amplified in BHK-21 cells as previously reported (Rieder et al., 2005).

### Construction of Deoptimized P2, P3 and P2/P3 Full Genome cDNA Clone

A derivative of the plasmid pA_24_Cru-WT was constructed to contain a unique *Nhe*I site in the 2A coding region (pA_24_Cru-*Nhe*I) (Supplementary Figures 1, 2). Deoptimized P2, P3, or P2/P3 clones were derived by subcloning codon modified sequences that were synthesized *de novo* (GenScript, Piscataway, NJ, United States) and designed using the method described by Burns et al. (2006) into pA_24_Cru-*Nhe*I backbone. Specifically, 1,517 bp *Nhe*I/*Mfe*I, 2,001 bp *Mfe*I/*Bam*HI, or 3512 bp *Nhe*I/*Bam*HI fragments containing P2 and/or P3 deoptimized sequences and negative antigenic markers in the 3B and 3Dpol regions as previously described (Uddowla et al., 2012) were substituted in pA_24_Cru-*Nhe*I. Modified plasmids were digested with *Swa*I for linearization and viral RNA were derived by *in vitro* transcription with T7 polymerase using MEGAscript T7 kit (Ambion) and purified with RNeasy (Qiagen) kit following the manufacturer’s directions. 10–20 μg of transcribed RNAs were electroporated into BHK-21 cells as previously described (Rieder et al., 2005) and after 24 h incubation at 37°C, cells were frozen for subsequent virus release and passage. Viruses were generated by passaging the virus at least five times at low multiplicity of infection (MOI = less than one) in BHK-21 cells. Recovered viruses were analyzed by standard Sanger sequencing and used for large scale preparation. Virus stocks were purified, concentrated by density gradient centrifugation in sucrose 10–50% (W/V) and sequenced again by the same sequencing method.

### CpG Sequence Analysis

Foot-and-mouth disease virus nucleotide sequences were downloaded from the National Center for Biotechnology Information (NCBI) website^1^ in FASTA format (accessed April 27th, 2020). Only sequences representing the full polyprotein ORF were extracted and analyzed alongside the deoptimized strains in SSE V1.4 for nucleotide and dinucleotide frequencies (Simmonds, 2012). CpG odds ratio were calculated using the equation CpG odds ratio = fCpG/fCfG.

### FMDV Cell Infections

Foot-and-mouth disease virus infections were performed in cultured cell monolayers at indicated multiplicity of infection (moi). As previously described (de los Santos et al., 2009) after 1 h adsorption at 37°C, unabsorbed virus was removed by washing the cells with a solution containing 150 mM NaCl in 20 mM morpholineethanesulfonic acid (MES) pH = 5.5, before adding MEM and proceeding with incubation at 37°C in 5% CO_2_. Infected cells were frozen at 1, 3, 6, and 24 h and virus titers were determined after thawing by plaque assay on BHK-21 cells. Plaques were counted at 48 hours post inoculation (hpi).

### Western Blotting

Cellular lysates were prepared as described previously (Medina et al., 2020). Proteins were analyzed by Western blotting using the following antibodies: anti-eIF4G from Bethyl Laboratories (Montgomery, TX, United States), anti-G3BP1 from Aviva Systems Biology (San Diego, CA, United States), anti-tubulin from NeoMarkers (Fremont, CA, United States). VP1 signal was detected using a rabbit polyclonal antibody developed in-house. Goat anti-rabbit immunoglobulin G (IgG) and goat anti-mouse IgG secondary antibodies conjugated to the enzyme horseradish peroxidase (HRP) were obtained from Pierce (Rockford, IL, United States). Protein samples were separated by SDS-PAGE and detected by WB using the specific antibodies indicated above and an ECL chemiluminescence Kit (Bio-Rad, Hercules, CA, United States). Images were acquired with the chemiluminescence digital imager Azure^®^ Imager c300.

### Animal Experiments

Animal experiments were conducted in the high-containment facilities of the Plum Island Animal Disease Center, conducted in compliance with the Animal Welfare Act (AWA), the 2011 Guide for Care and Use of Laboratory Animals, 2002 PHS Policy for the Humane Care and Use of Laboratory Animals, and United States Government Principles for Utilization and Care of Vertebrates Animals Used in Testing, Research and Training, as well as specific animal protocols reviewed and approved by the Institutional Animal Care and Use Committee (IACUC) of the Plum Island Animal Disease Center (USDA/APHIS/AC Certificate number: 21-F-0001; Protocol 204-14R for mice and 151-13R for swine).

#### Mice Experiment

C57BL/6 6–7-week-old female mice were purchased from Jackson Labs (Bar Harbor, ME, United States) and were acclimatized for 1 week. Groups of C57BL/6 mice (*n* = 6) were anesthetized with isoflurane (Webster Veterinary, Devens, MA, United States) and immediately infected subcutaneously (SC) in the left rear footpad with either 10^5^ pfu FMDV A_24_Cru (A24-WT) or 10^6^, or 10^7^ pfu each of FMDV A24-P2Deopt, A24-P3Deopt, or A24-P2/P3Deopt in 50 μl. Animals were monitored for 8 days. Viremia was determined by plaque assay on BHK-21 cells. 21 days after the initial inoculation, survivor mice were challenged (SC) in the right rear footpad with 5 × 10^5^ pfu of A24-WT and monitored for clinical development for 7 days and serum samples were collected for viremia detection. Also, serum samples were collected weekly to assess neutralizing antibody response.

#### Swine Experiments

In a first experiment, 23 Yorkshire gilts (5 weeks old and weighing approximately 18–23 kg each) were acclimated for 1 week and were randomly divided in five groups of four animals each and one group of three animals. In the five groups of four animals, three animals were inoculated intradermally in the heel bulb (IDHB) of the right hind foot with 10^6^ or 10^7^ pfu/animal of FMDV A24-P2Deopt or A24-P3Deopt or A24-P2/P3Deopt. The remaining animal of the group was a naïve animal housed in the same room for evaluation of contact transmission from directly inoculated animals.

In a second experiment, 16 swine were divided into four groups of four animals each. Three groups were IDHB inoculated as described above with 10^2^, 10^3^, or 10^5^ pfu/animal of FMDV A24-P2/P3Deopt. The remaining group was inoculated with 10^3^ pfu/animal of FMDV A24-WT.

Following each FMDV inoculation, clinical scores were evaluated daily for 7 days by determining the number of toes displaying FMD lesions and the presence of lesions in the snout and/or mouth. The maximum score considered was 17, and lesions restricted to the site of inoculation were not counted. The % of lymphocytes in the white cell population from whole blood collected in EDTA was measured for the first 7 days using a Hemavet cell counter (Drew Scientific-Erba Diagnostics, Miami Lakes, FL, United States). Samples of serum and nasal swab were collected the day of inoculation (baseline) and daily for 7 days after inoculation.

### Detection of Virus in Sera and Nasal Swabs

Mice and swine sera and swine nasal swabs were examined for the presence of virus by plaque assay on BHK-21 cells. Virus titers were expressed as log_10_ pfu/ml of serum or nasal swab secretions. The minimal detection level for this assay is 5 pfu/ml. In addition, FMDV RNA was detected by real-time RT-PCR (rRT-PCR) as previously described (Alejo et al., 2013). Cycle threshold (Ct) values were converted to RNA copies per milliliter (ml) using the equation derived from analysis of serial 10-fold dilutions of *in vitro* synthesized FMDV RNA of known concentration and expressed as the genome copy number per ml of serum or nasal swab.

### Evaluation of Humoral Immune Response

Neutralizing antibody titers were determined in mice or swine sera samples by end-point titration according to the method of Kärber (OIE, 2018). Antibody titers were expressed as the log_10_ value of the reciprocal of the dilution that neutralized 100 TCID_50_ in 50% of the wells. The presence of FMDV-specific IgM, IgG1, IgG2 antibodies was detected by an indirect double antibody sandwich assay as described previously (Diaz-San Segundo et al., 2012). Positive control sera for IgM or for IgG1 and IgG2 were obtained from a swine inoculated with virulent FMDV A_24_Cru at 7 or 21 days post-challenge (dpc), respectively. Positive control sera were chosen for their ability to generate a define signal within their respective isotype-specific assays. Pre-immune sera from the same animal was used as negative control sera for each assay. Detection of antibodies against non-structural polyprotein 3ABC in serum from A24-P2/P3Deopt and A24-WT inoculated animals was performed using commercial competitive enzyme-linked immunosorbent assay (cELISA) from Thermo Scientific-PrioCHECK^TM^ (Waltham, MA, United States) and VMRD^TM^ (Pullman, WA, United States) following manufactures instructions.

### Analysis of Cytokines in Serum

IFN-α, IL-1β, and IL-6 protein concentration was determined in sera from infected animals using ELISA. IFN-α was detected using mAbs K9 and F17 (PBL Interferon Source, Piscataway, NJ, United States) as previously described (Moraes et al., 2003). IL-1β and IL-6 Duo Set ELISAs (R&D Systems, Minneapolis, MN, United States) were performed following the manufacturer’s directions. All ELISA plates were developed with 3, 3′, 5, 5′, tetramethylbenzidine (TMB) peroxidase substrate solution from KPL (Gaithersburg, MD, United States). The absorbance at 450 nm was measured in an ELISA reader (Varioskan LUX, Thermo Scientific, Waltham, MA, United States). Cytokine concentrations were calculated based on the optical densities obtained with the standards and are expressed in relative levels with respect to the levels observed at 0 dpi.

### Data Analyses

Data handling, analysis, and graphic representations were performed using Prism 5.0 (GraphPad Software, San Diego, CA, United States) or Microsoft Excel (Microsoft, Redmond, WA, United States). Statistical significance was determined using Student’s t-, two-way ANOVA, or Gehan-Breslow-Wilcoxon tests.

## Results

### Synonymous Deoptimization of P2, P3, or P2/P3 Genomic Regions Results in Viable FMDV

Previous studies have shown that sequence deoptimization of the P1 genomic region is tolerated by FMDV (Diaz-San Segundo et al., 2016). In order to test if deoptimization would work for the highly conserved P2 and/or P3 regions of FMDV, we designed viral genomes in which codon usage was deoptimized by replacement with a non-preferred codon (Burns et al., 2006). Deoptimized sequences contained 320, 367, or 687 nucleotide substitutions throughout P2, P3, or P2/P3 coding regions, respectively (Supplementary Figure 1). In addition, negative antigenic markers were constructed in the 3B and 3Dpol viral proteins, (Uddowla et al., 2012; Supplementary Figure 2B). Modified sequences were synthetically obtained from a commercial supplier and subsequently replaced into the pA_24_-WT FMDV infectious cDNA clone (Rieder et al., 2005; Supplementary Figures 2A,B). CpG odds ratio analysis, which normalizes the CpG incidence to the expected CpG frequency, was performed on the full polyprotein ORF of all three deoptimized candidates, the parental sequence and 1055 independent full length FMDV ORF sequences downloaded from NCBI. Under no selective pressure, the CpG odd ratio should be 1, while values of 0.78 and 1.23 have previously been proposed as representing under or overrepresented CpG odds ratios within a particular RNA virus sequence (Cardon et al., 1994; Karlin et al., 1997; Cheng et al., 2013; Xia, 2020). The CpG odds ratio of A_24_Cru (0.80) was very similar to the average CpG odds ratio calculated for the ORF sequences downloaded from NCBI (0.81 ± 0.028) (range = 0.74–0.96) indicating that CpG dinucleotide is underrepresented in A_24_Cru and has been negatively selected considering the multiple FMDV genomes (Supplementary Figure 2C). In contrast, the A24-P2Deopt, A24-P3Deopt, and A24-P2/P3Deopt had CpG odds ratios of 0.96, 1.03, and 1.22, respectively, representing values larger than the average calculated from the database (Supplementary Figure 2C). It has previously been observed that CpG odds ratios increase when the GC % of a viral genome increase, however, the increased CpG odds ratio observed in the deoptimized mutants actually occurs with a slight decrease to the overall GC % of their genomes (Supplementary Figure 2C; Cheng et al., 2013).

**FIGURE 1:**
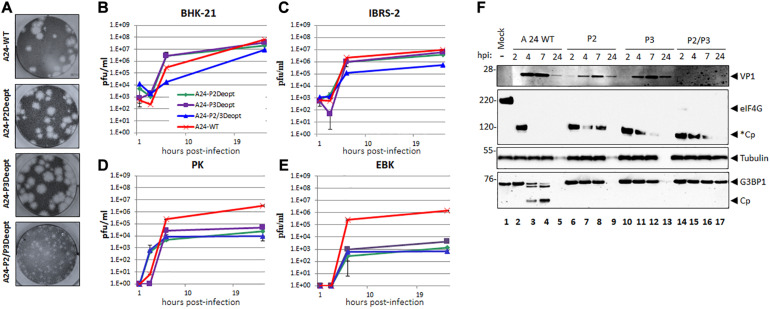
Plaque morphology and kinetics of growth in cell culture. **(A)** Plaque morphology of WT and deoptimized viruses was analyzed by plaque assay in BHK-21 cells. Cell monolayer were infected for 48 h in semisolid media followed by staining with crystal violet. Kinetics of growth in multiple cell lines: **(B)** BHK-21 and **(C)** IBRS-2 cells lines or **(D)** PK and **(E)** EBK cells were infected with FMDV A_24_Cru wild type (A24-WT) or deoptimized variants, A24-P2Deopt, A24-P3Deopt, and A24-P2/3Deopt at MOI = 2. Samples were taken at 1, 3, 6, and 24 hpi and virus titers were determined by plaque assay on BHK-21 cells. **(F)** LF-BKαVβ6 cells were mock infected or infected with either A24 WT, P2, P3, or P2/P3 deoptimized viruses and lysates were collected at 2, 4, 7, and 24 hpi. Proteins were resolved by SDS-PAGE and western blot was applied to detect FMDV VP1, eIF4G, G3BP1, and tubulin. Cleavage of eIF4G (220 kDa) generates a fragment of approximately 120 kDa (*Cp).

Viruses were derived after RNA electroporation and five blind passages at low MOI in BHK-21 cells. An extra passage at high MOI was required to prepare stocks of high titers (10^8^–10^9^ pfu/ml) for further characterization. Sanger sequencing indicated that consensus deoptimized viral sequences remained unchanged or have synonymous mutations. In BHK-21 cells viruses with P2 or P3 deoptimized sequences displayed a plaque morphology similar to WT, however, viruses containing both deoptimized P2 and P3 displayed a small plaque phenotype (Figure 1A), suggesting that the bigger the deoptimized region the greater the attenuation, as previously described for other picornaviruses (Coleman et al., 2008).

### FMDV A24 P2 and/or P3 Deoptimized Viruses Are Attenuated in Primary Cell Cultures

Growth kinetics of the A24-deoptimized viruses were analyzed in conventional cell lines used to propagate FMDV such as BHK-21 or IBRS-2, and in secondary cell cultured PK or EBK cells (Figures 1B–E). By 24 hpi, the three deoptimized viruses reached end point titers that were roughly similar to WT virus (∼ 10^7^ pfu/ml) in BHK-21 cells, although deoptimized A24-P2/P3Deopt grew at a slower rate (Figure 1B), consistent with the smaller plaque phenotype for this viral strain. A similar phenotype was detected in IBRS-2 cells but the end point titer of the A24-P2/P3Deopt virus was about one log lower (10^6^ pfu/ml) in comparison to the titers of A24-WT or A24-P2Deopt or A24-P3Deopt viruses (Figure 1C). Interestingly, in cells that have a functional innate immune system, such as secondary kidney cells derived from swine or bovines, all deoptimized viruses had an end point titer that was between 2 and 4 logs lower than the yield attained by WT virus (Figures 1D,E). These results indicated that all, specially A24P2/P3, mutant viruses were attenuated *in vitro*, with stronger phenotypes under selective pressures presumably driven by cellular immune responses.

During FMDV infection, rapid blockage of antiviral host responses mediated fundamentally by cleavage of cellular translation factor eIF4G and other factors including among several, stress granule scaffold protein G3BP1 (Devaney et al., 1988; Visser et al., 2019; Medina et al., 2020). To determine the dynamics of these cleavage events in cells infected with deoptimized viruses, the lysates from LFPKαvβ6 time course infection were subjected to Western blotting analysis (Figure 1F). A significant delay in the expression of the structural protein VP1 was detected for the P2 and P3 deoptimized viruses as compared to A24 WT at 4 and 7 hpi. Detection of VP1 was minimal in samples infected with P2/P3 deoptimized viruses which correlated with lower viral titers (6.6 × 10 e1pfu/ml) at those time points. Initial cleavage of eIF-4G was detected in all infected samples, as evidenced by the disappearance of the full length 220 kDa protein and the appearance of cleavage products of about ∼120 kDa. However, cleavage of these secondary products was delayed for all deoptimized viruses when compared to A24 WT. Interestingly, cleavage of G3BP1 cellular protein was not detected in any of the samples infected with deoptimized viruses while processing occurred as expected for the WT virus. These results suggest that the slower rate of eIF-4G full degradation and the inability to cleave G3BP1 by the deoptimized viruses may result in the induction of a stronger cellular antiviral response thereby leading to attenuation in host cells.

### Synonymous Deoptimization of P2 and/or P3 Coding Regions Results in Attenuation of FMDV in Mice

**FIGURE 2:**
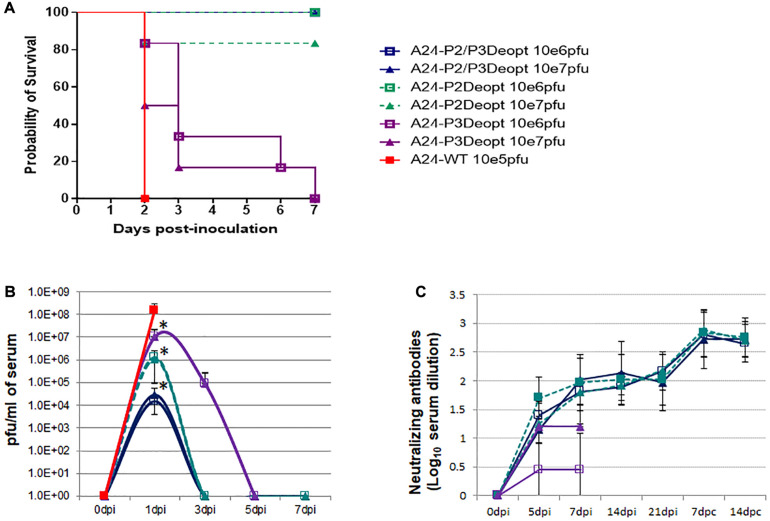
Deoptimized viruses are attenuated *in vivo* in mice. 6–7 weeks old female C57BL/6 mice (*n* = 6/group) were subcutaneously inoculated in the footpad with FMDV A24-deoptimized mutants, A24-P2/P3Deopt, A24-P2Deopt, or A24-P3Deopt at the indicated doses (plaque forming units –pfu-/animal). One group was inoculated with 1 × 10^5^ pfu/animal of A24-wild type (WT) as control. **(A)** Survival rates determined daily post inoculation. **(B)** Virus titers were measured in serum samples collected for 7 days post inoculation (dpi). **(C)** FMDV specific antibody neutralizing titers were measured in serum samples collected at 0, 5, 7, 14, and 21 dpi and at 7 and 14 days post-challenge (dpc). ^∗^*P* < 0.05.

Virulence of deoptimized viruses was determined in 6–7 week old female C57BL/6 mice by using the mouse FMD pathogenesis model described by Salguero et al. (2005) that showed consistency for multiple serotypes including A, C, and O (Diaz-San Segundo et al., 2013). Animals were inoculated with different doses of FMDV: A24-P2Deopt, A24-P3Deopt or A24-P2/P3Deopt or FMDV A24-WT, monitoring clinical signs, survival rate and the presence of virus in blood for a week after infection. As previously described (Diaz-San Segundo et al., 2014), animals inoculated with 10^5^ pfu of WT FMDV A24 developed clinical signs, including sluggishness and rough fur, and died by 2 days post inoculation (dpi) (Figure 2A). In contrast, deoptimized A24 viruses displayed different levels of attenuation depending on the virus variant and the dose. All animals inoculated with A24-P2/P3Deopt (independently of the dose), and animals inoculated with A24-P2Deopt at 10^6^ pfu survived, while 80% of the animals inoculated with 10^7^ pfu of A24-P2Deopt survived. Mice inoculated with A24-P3Deopt did not survive, however, disease progression was significantly slower as compared to WT FMDV A24 (*P* ≤ 0.05). Consistent with the survival data, animals inoculated with A24-P2/P3Deopt virus developed the lowest viremia levels (∼10^4^ pfu/ml), followed by A24-P2Deopt (∼10^6^ pfu/ml), A24-P3Deopt (∼10^7^ pfu/ml), and A24-WT (∼10^8^ pfu/ml) (Figure 2B). Viral titers of all the groups were statistically significantly different than the titers in the animals inoculated with A24-WT. As expected, following infection with the deoptimized strains, surviving mice developed protective levels of neutralizing antibodies with titers of about 2 Log_10_ starting at 7 dpi, reaching the peak by 14 dpi, with a strong anamnestic response after challenge with WT virus (Figure 2C).

### Synonymous Deoptimization of P2 and/or P3 Coding Regions Results in Attenuation of FMDV in Swine

The attenuation and protection observed in mice prompted us to evaluate virulence of A24-P2Deopt, A24-P3Deopt, and A24-P2/P3Deopt in swine, a natural host of FMDV. Groups of three pigs were inoculated IDHB in the rear heel bulb with 10^6^ or 10^7^ pfu/animal of A24-P2Deopt and A24-P2/P3Deopt. Given the relatively low attenuation observed in mice for the A24-P3Deopt virus in the mouse model, only one dose (10^6^ pfu/animal) was used. A naïve contact animal was included in each group and it remained in contact for the duration of the experiment.

**FIGURE 3:**
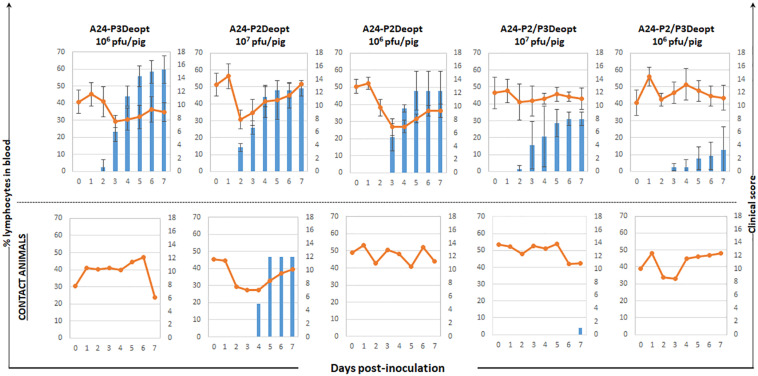
Clinical outcome in animals inoculated with A24 Deoptimized candidates at indicated doses. 18–23 kg castrated male Yorkshire swine (*n* = 3/group) were clinically monitored for 7 days after inoculation and samples of heparinized blood were collected daily. One contact animal was housed in direct contact to the animals inoculated with the different doses of indicated deoptimized candidates and subject to the same clinical evaluation and sampling regime. Average of clinical score (blue bars) and % of lymphocytes (orange line) are represented except for the contact animals that are represented individually.

As observed in Figure 3, animals inoculated with 10^6^ pfu/animal of A24-P3Deopt developed clinical signs of FMD, including vesicular lesions and lymphopenia, by 2 dpi reaching a high score (10–17 lesions) by 4 dpi, as it has previously been described for animals inoculated with 10 fold less A24-WT virus (Diaz-San Segundo et al., 2014). Animals inoculated with 10^7^ pfu/pig A24-P2Deopt also showed clinical signs starting at 2 dpi, although the overall clinical score was lower than that previously observed for A24-WT. As expected, severity of disease in animals inoculated with lower amounts of the same virus (10^6^ pfu/pig A24-P2Deopt) decreased as disease onset was delayed. Animals inoculated with A24-P2/P3Deopt displayed a significantly reduced severity of disease in a dose dependent manner; those inoculated with 10^7^ pfu/pig showed a maximum average clinical score of 8 without significant lymphopenia, starting at 2 dpi, and animals inoculated with 10^6^ pfu/pig showed a maximum average clinical score of 3, no lymphopenia and delayed disease onset (3 dpi) was detected. Interestingly, naïve animals maintained in contact with the animals directly inoculated with 10^6^ pfu/animal of either one of the three deoptimized FMDV variants did not develop clinical signs of disease (Figure 3).

**FIGURE 4:**
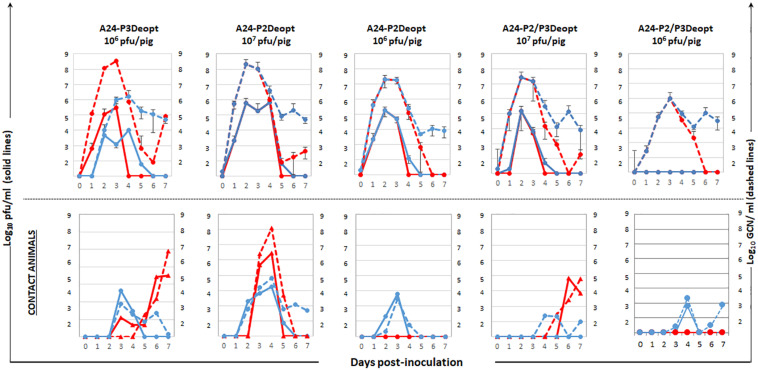
Virus detection in serum and nasal swabs in animals inoculated with different viruses at indicated doses. 18–23 kg castrated male Yorkshire swine (*n* = 3/group) inoculated with A24-P3Deopt, A24-P2Deopt, or A24-P2/P3Deopt at indicated doses of were sampled daily. One contact animal was housed in direct contact to the animals inoculated with the different doses of A24-P2/P3Deopt and subject to the same sampling regime. The amount of virus was detected by virus isolation in serum (red line) and nasal secretions (blue line) and by qPCR and expressed as genome copy numbers (GCN) per ml of serum (red dashed line) or per ml of nasal secretions (blue dashed line). Average of viral amounts are represented except for the contact animals that are represented individually.

Infectious virus was detected in sera and nasal swabs of all inoculated groups except for the swine inoculated with 10^6^ pfu/animal of A24-P2/P3Deopt, group with the least severe symptoms (Figure 4). However, viral RNA was detected in sera and nasal swabs of all inoculated animals. In the case of contact animals, all animals were positive for infectious virus and/or viral RNA in nasal swabs. Nevertheless, only animals that showed clinical disease were also positive in serum. Interestingly, the contact animal in the A24-P3Deopt that did not show clinical disease, did have detectable viremia (Figure 4).

This data indicates that the level of FMDV attenuation due to deoptimization varies depending on the extent and location of these changes in the viral genome. Clearly, there is a direct correlation between the length of deoptimized regions and the degree of attenuation, as previously described for other picornaviruses (Mueller et al., 2006).

### FMDV Deoptimized Mutants Elicit Strong Adaptive Humoral Immune Response in Swine

**FIGURE 5:**
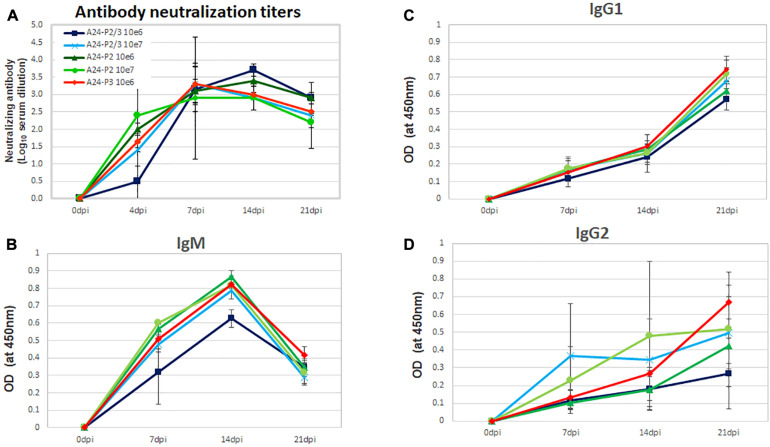
Determination of FMDV neutralizing antibodies or antibody isotype in the serum of animals inoculated with A24-P2, P3, or P2/P3Deopt. **(A)** The presence of FMDV neutralizing antibodies was evaluated in the sera of animals inoculated with A24-P2, P3, or P2/P3Deopt, by a microtiter neutralization assay on BHK-21 cells. Titers are reported as the log10 of the reciprocal of the highest dilution of serum that neutralized the virus in 50% of the wells. **(B–D)** Antibody isotype profiles in swine sera after inoculation. FMDV-specific IgM, IgG_1_, and IgG_2_ were detected by sandwich ELISA at 7, 14, and 21 dpi. Each data point represents the mean (±SD) of each group.

Natural FMDV infection induces a strong neutralizing antibody response as early as 4 dpi that ultimately clears the virus. Furthermore, attenuated FMDV strains can elicit the same levels of neutralizing antibodies as WT virus (Diaz-San Segundo et al., 2012). In the current experiment, we observed that despite the variation in attenuation observed for the different mutants, all animals developed similar levels of neutralizing antibodies consistent with values attained in animals inoculated with lower doses of WT virus (Figure 5A; Diaz-San Segundo et al., 2014). Furthermore, there was not a statistically significant difference in neutralizing antibody titers among the different groups at 7, 14, and 21 dpi, although at 4 dpi, lower levels were detected in animals inoculated with 10^6^ pfu/animal of A24-P2/P3Deopt.

In order to characterize the FMDV antibody response elicited after inoculation with deoptimized viruses, the specific immunoglobulin (Ig) isotype present in swine sera was determined. IgM was detected by 7 dpi in all inoculated animals, peaked at 14 days and declined by 21 days (Figure 5B). Parallel to observations for neutralizing antibodies, animals inoculated with10^6^ pfu/animal of A24-P2/P3Deopt showed lower IgM levels at 7 and 14 dpi, but by 21 dpi all animals displayed equivalent higher levels. On the other hand, the levels of IgG1 (Figure 5C) and IgG2 (Figure 5D) increased similarly in all inoculated animals with a peak at 21 dpi. Differences in the levels of either IgG1 or IgG2 among the groups, were not statistically significant. Together, these data indicate that inoculation of swine with deoptimized P2, P3, or P2/P3 FMDV mutants elicits a strong adaptive immune response.

### Systemic Cytokine Profile Elicited by the Different Deoptimized Mutants

**FIGURE 6:**
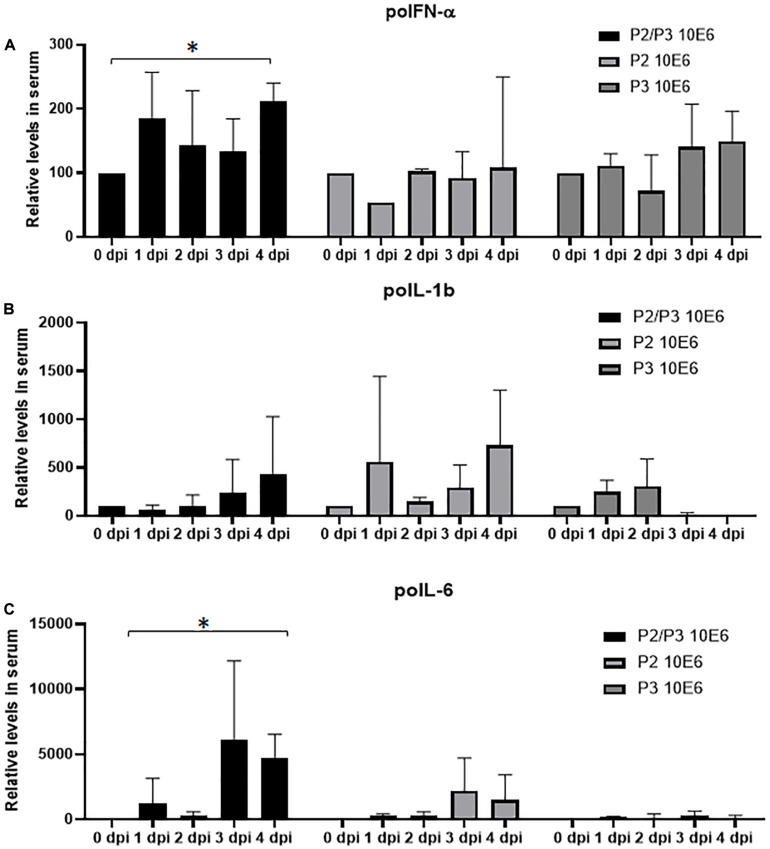
Cytokine protein profiles in serum after FMDV infection. Levels of IFN-α **(A)**, IL-1β **(B)**, and IL-6 **(C)** were detected by sandwich ELISA in the serum of inoculated animals with indicated doses, during the first 4 days after infection. Amount of protein is expressed as the relative level with respect to the amount observed at 0 dpi. Error bars represent the variation within the three animals from each group. ^∗^*P* < 0.05.

We have previously demonstrated that deoptimization of the FMDV P1 region induces a cellular innate immune response *in vitro* (Diaz-San Segundo et al., 2016). We have also demonstrated that during *in vivo* infection with FMDV WT there is a systemic reduction of some pro-inflammatory cytokines (Diaz-San Segundo et al., 2012). In this study we characterized systemic effects of the inoculation with the different deoptimized mutants by measuring the levels of several pro-inflammatory cytokines in sera. As observed in Figure 6, only animals inoculated with A24-P2/P3Deopt showed significant increase of poIFN-α and poIL-6 by 4 dpi, while animals inoculated with the other two mutants did not show significant differences. No statistically significant changes were detected in the levels of poIL-1b for any of the three variants.

### Lower Doses of A24-P2/P3Deopt_3__B__3__D_ Do Not Cause Disease in Swine

**FIGURE 7:**
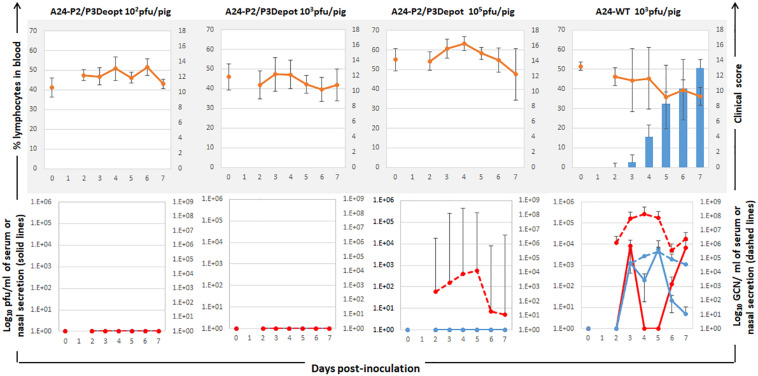
Clinical outcome and virus shedding in animals inoculated with different doses of A24-P2/P3Deopt and A_24_Cru wild type (A24-WT). 18–23 kg castrated male Yorkshire swine (*n* = 4/group) were inoculated with 10^2,^ 10^3^, or 10^5^ pfu/animal of FMDV A24-P2/P3Deopt or 10^3^ pfu/animal of A24-WT as control group. Animals were monitored for 7 days. **Upper graphs:** average of clinical score (blue bars) and % of lymphocytes (orange line) were determined daily. **Lower graphs:** The amount of virus was detected by virus isolation in serum (red line) and nasal secretions (blue line) and by qPCR and expressed as genome copy numbers (GCN) per ml of serum (red dashed line) or per ml of nasal secretions (blue dashed line).

Given that the highest levels of attenuation were achieved in the A24-P2/P3Deopt variant *in vitro* and *in vivo*, a second swine experiment was performed to determine whether lower (less than 10^6^ pfu/animal) viral doses would not cause disease and at the same time, induce immune responses that could provide protection against challenge with WT virus. Groups of four pigs were inoculated with 10^2^, 10^3^ of 10^5^ of A24-P2/P3Deopt virus and one group was inoculated with 10^3^ pfu/animal of A24-WT as control. As seen in Figure 7, none of the animals inoculated with A24-P2/P3Deopt virus developed clinical signs by 7 dpi. In contrast, all swine inoculated with 10^3^ pfu/animal of A24-WT developed disease. In the control group inoculated with fully virulent A24-WT, three of the four inoculated animals reached high scores (15–17 lesions, Max = 17) by 5–7 dpi despite the low dose used (10^3^ pfu/animal), resembling the kinetics observed in other swine experiment that used 100x higher doses (10^5^ pfu/animal; Diaz-San Segundo et al., 2014). None of the animals inoculated with A24-P2/P3Deopt developed vesicular lesions, although one animal inoculated with 10^5^ of A24-P2/P3Deopt had detectable lymphopenia by 7 dpi followed by a rapid recovery (data not shown). Consistently, virus or viral RNA were detected in serum and nasal swabs of all animals inoculated with A24WT virus, while no virus was detected in serum or nasal secretions in all animals inoculated with A24-P2/P3Deopt (Figure 7). Interestingly, in the group inoculated with 10^5^ of A24-P2/P3Deopt one animal was positive for viral RNA in serum and showed late lymphopenia (data not shown).

### Inoculation of Swine With Low Doses of A24-P2/P3Deopt Does Not Protect Against Challenge With WT Virus

**FIGURE 8:**
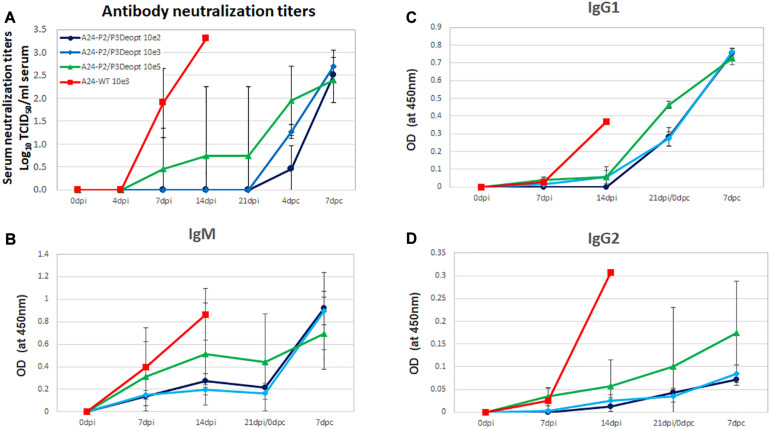
Determination of FMDV neutralizing antibodies and isotypes in the serum of animals inoculated with A24-P2/P3Deopt or A_24_Cru wild type (A24-WT). **(A)** Presence of FMDV neutralizing antibodies was evaluated by a microtiter neutralization assay on BHK-21 cells in sera of animals inoculated with different doses of A24-P2/P3Deopt or A24-WT at the indicated time points after inoculation or challenge. Titers are reported as the log_10_ of the reciprocal of the highest dilution of serum that neutralized the virus in 50% of the wells. **(B–D)** Antibody isotype profiles in swine sera after inoculation. FMDV-specific IgM, IgG_1_, and IgG_2_ were detected by sandwich ELISA at indicated time points. Each data point represents the mean (±SD) of each group.

Analysis of specific neutralizing antibody titers throughout the experiment showed a relatively mild response that was only detectable in the group inoculated with 10^5^ pfu of A24-P2/P3Deopt. Neutralizing antibody titers were undetectable in animals inoculated with lower doses (Figure 8A). As expected, animals inoculated with FMDV A24-WT developed neutralizing antibodies starting at 7 dpi but showed a slight delay as compared to previous experiments in which animals were inoculated with higher doses of the same lot of WT virus (Diaz-San Segundo et al., 2014). Similarly, analysis of the different isotypes showed very low levels of either IgM (Figure 8B), IgG1 (Figure 8C), and IgG2 (Figure 8D) as compared to the levels elicited by inoculation with WT or with higher doses of A24-P2/P3Deopt (Figures 5B–D). Detection of antibodies against non-structural proteins, 3ABC cELISA, was negative in sera of all A24-P2/P3 inoculated animals at 21 dpi, except for the one animal in which viral RNA had been detected in serum by real time PCR. As expected, animals inoculated with A24-WT were all 3ABC positive starting at 14 dpi (data not shown).

**FIGURE 9:**
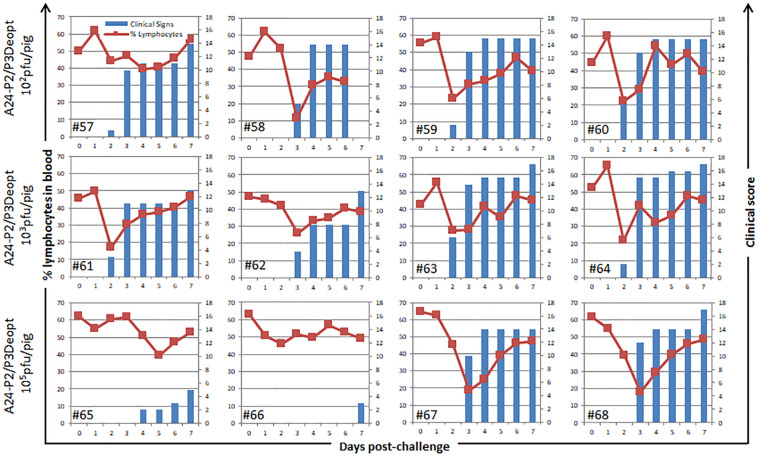
Clinical outcome in animals challenged with A_24_Cru (A24-WT) after previous inoculation with A24-P2/P3Deopt. 18–23 kg castrated male Yorkshire swine (*n* = 4/group) were challenged with 10^5^ plaque forming units (pfu) of FMDV A24-WT. Animals were monitored for 7 days and samples of heparinized blood were collected daily. Clinical score (blue bars) and % of lymphocytes (green line) for each animal are represented for each individual animal.

Challenge of these animals with homologous FDMV A24-WT showed that none of the animals inoculated with the lowest doses (10^2^ and 10^3^ pfu) of A24-P2/P3Deopt were protected against clinical signs of disease, while animals inoculated with 10^5^ of A24-P2/P3Deopt were partially protected (Figure 9). Interestingly, the levels of neutralizing antibodies, IgM and IgG1 showed an anamnestic response after challenge, indicating that animals had been primed by the inoculation of low doses of the deoptimized variant.

## Discussion

In a previous study, as a proof of concept, we used the SAVE technology to show the feasibility of creating a codon-pair deoptimized P1 FMDV A_12_ strain that was highly attenuated in virulence, both, *in vitro* and *in vivo*, in pigs (Diaz-San Segundo et al., 2016). In this study, we focused on codon deoptimization of highly conserved non-structural genes encoded in P2 and/or P3 regions of a more environmentally relevant A_24_Cru field FMDV strain. The deoptimization algorithm targeted specific regions circumventing areas in which secondary and tertiary RNA structures had been predicted to avoid possible interference with virus replication. The hypothesis behind this approach was the creation of a viral strain that could display attenuating factors within the P2/P3 highly conserved coding regions serving as the basis for development into FMDV LAV candidates. Accordingly, the vector was created with convenient restriction sites added to the 5′ and 3′ regions of the variable P1 region to allow for strain/serotype specific capsid swapping (Uddowla et al., 2012). Three viable strains of virus (A24-P2Deopt, A24-P3Deopt, and A24-P2/P3Deopt) that maintained the mutations were generated.

Our data demonstrates that the deoptimized FMDV viruses behaved, in cell culture, similarly to other previously reported attenuated FMDV strains obtained by different methodologies (Cottral et al., 1966; Mason et al., 1997; de los Santos et al., 2009; Diaz-San Segundo et al., 2016). In general, those strains had an aberrant growth rate, a decrease in end point titer, an increase in specific infectivity and produced small size plaques. The specific level of attenuation achieved in our experiments was dependent on both, the particular deoptimized region/s and the cell type used in the analysis. As previously observed for many attenuated mutants, the highest levels of attenuation were detected in cells (EBK and PK) that contain an intact innate immune system. Interestingly, *in vivo*, the FMDV A24-P2/and/or P3 deoptimized viruses were attenuated in both mice and in pigs and the level of attenuation was dependent on the dose and the specific deoptimized region.

The cumulative data suggests that the simultaneous deoptimization of both P2 and P3 achieved the highest levels of attenuation. These observations are in concurrence with data observed in IAV, RSV, DENV, and poliovirus using similar technologies, or in HAV where “natural deoptimization” has evolved to make tRNA pools more available for the virus; in the simplest sense, attenuation seems to be dependent on the number of deoptimized codons, and on the specific regions or gene/s (Burns et al., 2009; Aragonès et al., 2010; Stauft et al., 2018; Le Nouën et al., 2019, 2020). Mechanistically, the cause of attenuation seems to be more complicated and less definitive. Previous research has outlined that codon or codon-pair deoptimization can result in an increase of immunogenic molecules (CpG and UpA), decreased stability of the genome, loss of RNA secondary structures, a decrease in the rate of translation, co-translational misfolding, and a decreased rate of replication (Burns et al., 2009; Tulloch et al., 2014; Yu et al., 2015; Le Nouën et al., 2019; Groenke et al., 2020). Apparently, for FMDV, codon deoptimization of the NS coding regions affected the rate of replication/translation, and also inhibited the ability of the virus to cleave cellular host factors such as G3BP1 involved in modulation of antiviral responses. All these events may have contributed to the observed levels of viral attenuation. These results are supported by the evidence of reduced virulence, in particular in primary cells that have an intact innate immune system such as EBK and PK (Chinsangaram et al., 1999). Consistent with these results, increased expression of poIFN-α and poIL-6 was detected in pigs inoculated with A24-P2/P3Deopt. Although the increase of CpG presumably led to a more active innate immune system response, the contribution of other factors (genome instability, cell-specific decreased translation, etc.) remains to be examined. To this end, the exact mechanism that led to the attenuation of these viruses, still remains unclear, and warrants further studies.

The levels of attenuation in a mouse model resembled those observed *in vitro*, in which A24-P2/P3Deopt was the most attenuated of the three deoptimized strains. Furthermore, induction of the adaptive immune response successfully protected mice against challenge with virulent FMDV. Unfortunately, unlike in mice, A24-P2/P3Deopt was too attenuated in swine and did not induce a strong antibody-mediated protective response. All animals inoculated with A24-P2/P3Deopt at doses that were not sufficient to cause disease, were not protected against subsequent challenge with wild type A_24_Cru FMDV. In general for FMD, high antibody production induced by vaccination correlates with protection (Pay and Hingley, 1987; McCullough et al., 1992), although protection against challenge has been observed for experimental vaccines with relatively low levels of neutralizing antibodies, presumably mediated by cellular immune responses (Moraes et al., 2011). The average levels of serum neutralizing antibodies elicited in the group inoculated with the highest dose of A24-P2/P3Deopt (10^5^ pfu/pig) that did not cause clinical symptoms, was still lower than the levels of antibodies induced by the previously reported A12-P1Deopt strain (10^5^ pfu/pig) (Diaz-San Segundo et al., 2016), and also A12-SAP (10^5^ pfu/pig), an attenuated FMDV strain with a double amino acid substitution in viral Lpro (Diaz-San Segundo et al., 2012). Lack of detectable viable virus in serum could contribute to the overall lack of antibody induction; however, previous studies have shown induction of neutralizing antibodies without viremia (Diaz-San Segundo et al., 2016). In recent studies with other deoptimized vaccine candidates for other viral disease, i.e., DENV, Stauft et al. (2019a) showed that promising candidates initially tested in mice, were also too attenuated to induce a protective immune response when tested in more relevant species (Rhesus macaque). Accordingly, if deoptimization in the P2 and/or P3 regions is to be used as a vaccine backbone, further refinement of the deoptimization parameters to “de-attenuate” the strains used in combination with other well-established attenuating mutations in FMDV NS proteins (Diaz-San Segundo et al., 2012) might be required. In fact, this strategy has recently been applied to develop a new vaccine platform for poliovirus which is currently on Phase II study in adults, toddlers, and infants (Konopka-Anstadt et al., 2020).

Our results demonstrate that the use of deoptimization technology in FMDV NS protein genome region is a viable approach to develop attenuated FMDV strains. At the same time, this platform offers capsid swapping capabilities, and DIVA markers. Most importantly, codon deoptimization offers the advantage of allowing the artificial “erasing” of RNA sequences or motifs that might be involved in recombination, thus limiting the possibility of reversion to virulence in case of exposure to field virulent strains. On this topic, studies have shown that hot spots for FMDV genomic recombination may lie in RNA sequences coding for NS proteins (Carrillo et al., 2005; Brito et al., 2018; Ferretti et al., 2020).

The advantage of using LAV candidates for FMD, is the potential of inducing a rapid and presumably long-lasting immune response. In this regard, we previously demonstrated that swine inoculated with the FMDV SAP mutant were protected swine against WT virus challenge, as early as 2 days after vaccination, in the absence of systemic detectable neutralizing antibodies (Diaz-San Segundo et al., 2012). Attenuation by codon or codon pair deoptimization, is the result of shuffling a high number of nucleotides in a relatively small genome without changing the amino acid sequence. Thus, the probability of reversion to virulence under selective immune pressure is unlikely.

Of outmost importance, the long-term sequence stability of the P2 and/or P3 deoptimized regions remains to be determined. Although the Sanger sequencing method used in this manuscript initially indicated there is certain stability of the modified sequences, it should not be overstated, as it only determines the consensus sequence of the virus quasispecies. Further studies that pair, *in vitro* infections at low multiplicity, thus encouraging multiple rounds of replication and consequent accumulation of mutations, together with, in depth analysis using next generation sequencing, should be performed as a measure of evaluating the genomic stability of deoptimized viruses, both *in vitro* and *in vivo*. This topic is of outmost importance considering that compensatory mutations may also accumulate during large scale vaccine preparation leading to possible genotypes associated with increased virulence and decreased vaccine safety.

In the current studies, as well as in our previous work (Diaz-San Segundo et al., 2016), pigs were used for evaluation of the pathogenesis of deoptimized strains in a natural host species. However, bovids are perhaps the species of most interest for FMD, given their commercial value in the livestock industry and the fact that they serve as reservoirs of FMDV in the wild. As mentioned in the introduction previous work with a FMDV leaderless virus, showed high degree of attenuation, both, in cattle and pigs (Mason et al., 1997; Chinsangaram et al., 1998). In contrast, inoculation of pigs with an intertypic chimeric leaderless FMDV variant caused low, but detectable disease in pigs (Almeida et al., 1998). However, the newly developed leaderless vaccine candidate, FMDVLL_3__B__3__D,_ was very attenuated in both, cattle and pigs, providing optimal conditions for development as a safe inactivated FMD vaccine platform (Uddowla et al., 2012; Hardham et al., 2020). All together, these results highlight the importance of evaluating any LAV vaccine candidate in more than one host species if applicable, given the differential species-specific immune response and pathogenesis.

Another important aspect in the FMD vaccine field is the establishment of carrier state described only for convalescent or vaccinated cattle. It is known that over 50% of bovines that are exposed to FMDV usually become asymptomatic persistently infected/carriers (reviewed by Stenfeldt and Arzt, 2020). Commercially available FMD vaccines, such as inactivated whole virus, or recently developed vector delivered virus like particles, have failed to preventing persistence. Except for limited proof of concept studies with the FMDV SAP mutant that were performed in pigs (Diaz-San Segundo et al., 2012), no LAV candidate has been evaluated for FMD in the last 30 years, therefore there are no studies reporting efficacy in preventing persistence. One interesting point, however, is to consider that animals may become persistently infected only if their upper respiratory tract is exposed to live virus (Sutmoller et al., 1968). Remarkably, the same authors reported that no FMDV carrier state could be detected when animals were intramuscularly infected with the same virus, emphasizing the critical involvement of the bovine upper respiratory tract in the establishment of primary and persistent FMDV infection. These studies suggest that, as long as a LAV candidate is parenterally inoculated, vaccinated animals will unlikely become carriers. However, further experimentation is required to confirm this hypothesis and verify reproducibility, if any, for multiple virus serotypes.

It appears, however, that the combination of the deoptimization technology with the rational design of mutations targeting FMDV virulence factors may render viruses with the adequate level of attenuation necessary to increase the safety of novel vaccine candidates. As demonstrated for poliovirus (Konopka-Anstadt et al., 2020; Yeh et al., 2020), a multi-type vaccine approach might be needed as a path to eradication of FMD, a disease that has jeopardized economic sustainability and development of many countries for over a century in a rapidly changing World.

## Data Availability Statement

The original contributions presented in the study are included in the article/Supplementary Material, further inquiries can be directed to the corresponding author.

## Ethics Statement

The animal study was reviewed and approved by the Institutional Animal Care and Use Committee (IACUC) of the Plum Island Animal Disease Center (USDA/APHIS/AC Certificate number: 21-F-0001; Protocol 204-14R for mice and 151-13R for swine).

## Author Contributions

FD-S designed the studies, performed the experiments, and analyzed the data. GM performed the experiments and analyzed the data. ES analyzed the data. AK, ER-M, and PA performed the experiments. SM developed the theory and designed the deoptimized viral genomes. ER contributed to the overall design of the experiments. TS conceived and directed the project. FD-S, GM, and TS wrote the manuscript. All authors discussed the experimental design and results and contributed to the writing and editing of the final version of the manuscript.

## Conflict of Interest

SM is an employee at Codagenix Inc. The remaining authors declare that the research was conducted in the absence of any commercial or financial relationships that could be construed as a potential conflict of interest.

## Footnotes

1 http://www.ncbi.nlm.nih.gov

## Supplementary Material

Supplementary Figure 1 Sequences of A_24_Cru with deoptimized **(A)** P2, **(B)** P3, and **(C)** P2/P3. Underlined are *Nhe*I, *Mfe*I, and *Bam*HI restriction sites.

Supplementary Figure 2 Generation of deoptimized FMDV and sequence analysis. **(A)** Schematics of A_24_Cru wild type (WT) infectious clone with a unique added *Nhe*I site. Relevant restriction sites used for cloning are depicted (*Nhe*I, *Mfe*I, and *Bam*HI). **(B)** *Nhe*I/*Mfe*I, *Mfe*I/*Bam*HI of *Nhe*I/*Bam*HI fragments. containing deoptimized codons were synthesized and respectively replaced in pA_24_CruN*he*I. Synthesized fragments contained DIVA markers including a small deletion in 3B and amino acid substitutions in 3B and 3D. **(C)** Full polyprotein ORF from sequences obtained from National Center for Biotechnology Information (NCBI) website (http://www.ncbi.nlm.nih.gov) (*n* = 1055) were analyzed alongside the deoptimized strains in SSE V1.4 for nucleotide and dinucleotide frequencies (Simmonds, 2012). CpG odds ratio were calculated using the equation CpG odds ratio = fCpG/fCfG.

## References

[B1] Alejo D. M. Moraes M. P. Liao X. Dias C. C. Tulman E. R. Diaz-San Segundo F. (2013). An adenovirus vectored mucosal adjuvant augments protection of mice immunized intranasally with an adenovirus-vectored foot-and-mouth disease virus subunit vaccine. *Vaccine* 31 2302–2309. 10.1016/j.vaccine.2013.02.060 23499593

[B2] Almeida M. R. Rieder E. Chinsangaram J. Ward G. Beard C. Grubman M. J. (1998). Construction and evaluation of an attenuated vaccine for foot-and-mouth disease: difficulty adapting the leader proteinase-deleted strategy to the serotype O1 virus. *Virus Res.* 55 49–60. 10.1016/s0168-1702(98)00031-8 9712511

[B3] Aragonès L. Guix S. Ribes E. Bosch A. Pintó R. M. (2010). Fine-tuning translation kinetics selection as the driving force of codon usage bias in the hepatitis A virus capsid. *PLoS Pathog.* 6:e1000797. 10.1371/journal.ppat.1000797 20221432 PMC2832697

[B4] Brito B. Pauszek S. J. Hartwig E. J. Smoliga G. R. Vu L. T. Dong P. V. (2018). A traditional evolutionary history of foot-and-mouth disease viruses in Southeast Asia challenged by analyses of non-structural protein coding sequences. *Sci. Rep.* 8:6472. 10.1038/s41598-018-24870-6 PMC5915611 29691483

[B5] Burns C. C. Campagnoli R. Shaw J. Vincent A. Jorba J. Kew O. (2009). Genetic inactivation of poliovirus infectivity by increasing the frequencies of CpG and UpA dinucleotides within and across synonymous capsid region codons. *J. Virol.* 83 9957–9969. 10.1128/jvi.00508-09 19605476 PMC2747992

[B6] Burns C. C. Shaw J. Campagnoli R. Jorba J. Vincent A. Quay J. (2006). Modulation of poliovirus replicative fitness in HeLa cells by deoptimization of synonymous codon usage in the capsid region. *J. Virol.* 80 3259–3272. 10.1128/jvi.80.7.3259-3272.2006 16537593 PMC1440415

[B7] Cardon L. R. Burge C. Clayton D. A. Karlin S. (1994). Pervasive CpG suppression in animal mitochondrial genomes. *PNAS* 9 3799–3803. 10.1073/pnas.91.9.3799 8170990 PMC43669

[B8] Carrillo C. Tulman E. R. Delhon G. Lu Z. Carreno A. Vagnozzi A. (2005). Comparative genomics of foot-and-mouth disease virus. *J. Virol.* 79 6487–6504. 15858032 10.1128/JVI.79.10.6487-6504.2005 PMC1091679

[B9] Cheng X. Virk N. Chen W. Ji S. Ji S. Sun Y. (2013). CpG usage in RNA viruses: data and hypotheses. *PLoS One* 8:e74109. 10.1371/journal.pone.0074109 24086312 PMC3781069

[B10] Chinsangaram J. Mason P. W. Grubman M. J. (1998). Protection of swine by live and inactivated vaccines prepared from a leader proteinase-deficient serotype A12 foot-and-mouth disease virus. *Vaccine* 16 1516–1522. 10.1016/s0264-410x(98)00029-2 9711798 PMC7172646

[B11] Chinsangaram J. Piccone M. E. Grubman M. J. (1999). Ability of foot-and-mouth disease virus to form plaques in cell culture is associated with suppression of alpha/beta interferon. *J. Virol.* 73 9891–9898. 10.1128/jvi.73.12.9891-9898.1999 10559301 PMC113038

[B12] Coleman J. R. Papamichail D. Skiena S. Futcher B. Wimmer E. Mueller S. (2008). Virus attenuation by genome-scale changes in codon pair bias. *Science* 320 1784–1787. 10.1126/science.1155761 18583614 PMC2754401

[B13] Cottral G. E. Patty R. E. Gailiunas P. C. Scott F. W. (1966). Relationship of foot-and-mouth disease virus plaque size on cell cultures to infectivity for cattle by intramuscular inoculation. *Archiv. F. Virusforschung.* 18 276–293. 10.1007/bf01250142

[B14] de los Santos T. Diaz-San Segundo F. Rodriguez L. L. (2018). The need for improved Vaccines agaisnt foot-and-mouth disease. *Curr. Opin. Virol.* 29 16–25. 10.1016/j.coviro.2018.02.005 29544088

[B15] de los Santos T. Diaz-San Segundo F. Zhu J. Koster M. Dias C. C. Grubman M. J. (2009). A conserved domain in the leader proteinase of foot-and-mouth disease virus is required for proper subcellular localization and function. *J. Virol.* 83 1800–1810. 10.1128/jvi.02112-08 19052079 PMC2643771

[B16] Devaney M. A. Vakharia V. N. Lloyd R. E. Ehrenfeld E. Grubman M. J. (1988). Leader protein of foot-and-mouth disease virus is required for cleavage of the p220 component of the cap-binding protein complex. *J. Virol.* 62 4407–4409. 10.1128/jvi.62.11.4407-4409.1988 2845152 PMC253884

[B17] Diaz-San Segundo F. Dias C. C. Moraes M. P. Weiss M. Perez-Martin E. Owens G. (2013). Venezuelan equine encephalitis replicon particles can induce rapid protection against foot-and-mouth disease. *J. Virol.* 87 5447–5460. 10.1128/jvi.03462-12 23468490 PMC3648198

[B18] Diaz-San Segundo F. Dias C. C. Moraes M. P. Weiss M. Perez-Martin E. Salazar A. M. (2014). Poly ICLC increases the potency of a replication-defective human adenovirus vectored foot-and-mouth disease vaccine. *Virology* 468-470 283–292. 10.1016/j.virol.2014.08.012 25216089

[B19] Diaz-San Segundo F. Medina G. N. Ramirez-Medina E. Velazquez-Salinas L. Koster M. Grubman M. J. (2016). Synonymous deoptimization of foot-and-mouth disease virus causes attenuation in vivo while inducing a strong neutralizing antibody response. *J Virol.* 90 1298–1310. 10.1128/jvi.02167-15 26581977 PMC4719607

[B20] Diaz-San Segundo F. Weiss M. Pérez-Martín E. Dias C. C. Grubman M. J. de los Santos T. (2012). Inoculation of swine with foot-and-mouth disease SAP-mutant virus induces early protection against disease. *J. Virol.* 86 1316–1327. 10.1128/jvi.05941-11 22114339 PMC3264347

[B21] Doel T. R. (2003). FMD vaccines. *Virus Res.* 91 81–99. 10.1016/s0168-1702(02)00261-7 12527439

[B22] Domingo E. Escarmís C. Baranowski E. Ruiz-Jarabo C. M. Carrillo E. Núñez J. I. (2003). Evolution of foot-and-mouth disease virus. *Virus Res.* 91 47–63. 12527437 10.1016/s0168-1702(02)00259-9

[B23] Eschbaumer M. Dill V. Carlson J. C. Arzt J. Stenfeldt C. Krug P. W. (2020). Foot-and-mouth disease virus lacking the leader protein and containing two negative DIVA markers (FMDV LL3B3D A 24) is highly attenuated in pigs. *Pathogens* 9:129. 10.3390/pathogens9020129 32079312 PMC7168223

[B24] Ferretti L. Perez-Martin E. Zhang F. Maree F. de Klerk-Lorist L.-M. van Schalkwykc L. (2020). Pervasive within-host recombination and epistasis as major determinants of the molecular evolution of the foot-and-mouth disease virus capsid. *PLoS Pathog.* 16:e1008235. 10.1371/journal.ppat.1008235 31905219 PMC6964909

[B25] Glynn I. Glynn J. (2004). *The Life and Death of Smallpox.* Cambridge: Cambridge University Press.

[B26] Greenwood B. (2014). The contribution of vaccination to global health: past, present and future. *Phil. Trans. R. Soc. B* 369:20130433. 10.1098/rstb.2013.0433 24821919 PMC4024226

[B27] Groenke N. Trimpert J. Merz S. Conradie A. M. Wyler E. Zhang H. (2020). Mechanism of virus attenuation by codon pair deoptimization. *Cell Rep.* 31:107586. 10.1016/j.celrep.2020.107586 32348767

[B28] Grubman M. J. Baxt B. (2004). Foot-and-mouth disease. *Clin. Microbiol. Rev.* 17 465–493. 15084510 10.1128/CMR.17.2.465-493.2004 PMC387408

[B29] Grubman M. J. Moraes M. Schutta C. Barrera J. Neilan J. Ettyreddy D. (2010). Adenovirus serotype 5-vectored foot-and-mouth disease subunit vaccines: the first decade. *Future Virol.* 5 51–64. 10.2217/fvl.09.68

[B30] Hardham J. M. Krug P. W. Pacheco J. M. Thompson J. Dominowski P. Moulin M. (2020). Novel foot-and-mouth disease vaccine platform: formulations for safe and DIVA-compatible FMD vaccines with improved potency. *Front. Vet. Sci.* 7:554305. 10.3389/fvets.2020.554305 33088833 PMC7544895

[B31] Ikemura T. (1981). Correlation between the abundance of *Escherichia coli* transfer RNAs and the occurrence of the respective codons in its protein genes: a proposal for a synonymous codon choice that is optimal for the *E. coli* translational system. *J. Mol. Biol.* 151 389–409. 10.1016/0022-2836(81)90003-6 6175758

[B32] Karlin S. Mrázek J. Campbell A. M. (1997). Compositional biases of bacterial genomes and evolutionary implications. *J. Bacteriol.* 179 3899–3913. 10.1128/jb.179.12.3899-3913.1997 9190805 PMC179198

[B33] Kloc A. Diaz-San Segundo F. Schafer E. A. Rai D. K. Kenney M. de Los Santos T. (2017). Foot-and-mouth disease virus 5’-terminal S fragment is required for replication and modulation of the innate immune response in host cells. *Virology* 512 132–143. 10.1016/j.virol.2017.08.036 28961454

[B34] Konopka-Anstadt J. L. Campagnoli R. Vincent A. Shaw J. Wei L. Wynn N. T. (2020). Development of a new oral poliovirus vaccine for the eradication end game using codon deoptimization. *NPJ Vacc.* 5:26. 10.1038/s41541-020-0176-7 32218998 PMC7083942

[B35] LaRocco M. Krug P. W. Kramer E. Ahmed Z. Pacheco J. M. Duque H. (2015). Correction for LaRocco et al., a continuous bovine kidney cell line constitutively expressing bovine αVβ6 integrin has increased susceptibility to foot-and-mouth disease virus. *J. Clin. Microbiol.* 53:755. 10.1128/jcm.03220-14 25617444 PMC4298512

[B36] Le Nouën C. Brock L. G. Luongo C. McCarty T. Yang L. Mehedi M. (2014). Attenuation of human respiratory syncytial virus by genome-scale codon-pair deoptimization. *Proc. Natl. Acad. Sci. U.S.A.* 111 13169–13174. 10.1073/pnas.1411290111 25157129 PMC4246931

[B37] Le Nouën C. Collins P. L. Buchholz U. J. (2019). Attenuation of human respiratory viruses by synonymous genome recoding. *Front. Immunol.* 10:1250. 10.3389/fimmu.2019.01250 31231383 PMC6558635

[B38] Le Nouën C. Luongo C. L. Yang L. Mueller S. Wimmer E. DiNapoli J. M. (2020). Optimization of the codon pair usage of human respiratory syncytial virus paradoxically resulted in reduced viral replication in vivo and reduced immunogenicity. *J Virol.* 94 e1296–e1219. 10.1128/JVI.01296-19 PMC6955273 31666376

[B39] Lee S. Y. Lee Y. J. Kim R. H. Park J. N. Park M. E. Ko M. K. (2017). Rapid engineering of foot-and-mouth disease vaccine and challenge viruses. *J. Virol.* 91:e155-17. 10.1128/JVI.00155-17 PMC5533925 28566375

[B40] Li P. Ke X. Wang T. Tan Z. Luo D. Miao Y. (2018). Zika virus attenuation by codon pair deoptimization induces sterilizing immunity in mouse models. *J. Virol.* 92 e701–e718. 10.1128/JVI.00701-18 PMC6096834 29925661

[B41] Mahy B. W. (2004). “Overview of foot-and-mouth disease and its impact as a re-emergent viral infection,” in *Foot-and-Mouth Disease: Current Perspectives*, eds Sobrino F. Domingo E. (Boca Raton, FL: CRC Press), 437–446. 10.1201/9780429125614-17

[B42] Martrus G. Nevot M. Andres C. Clotet B. Martinez M. A. (2013). Changes in codon-pair bias of human immunodeficiency virus type 1 have profound effects on virus replication in cell culture. *Retrovirology* 10:78. 10.1186/1742-4690-10-78 23885919 PMC3726367

[B43] Mason P. W. Piccone M. E. McKenna T. S. Chinsangaram J. Grubman M. J. (1997). Evaluation of a live-attenuated foot-and-mouth disease virus as a vaccine candidate. *Virology* 227 96–102. 10.1006/viro.1996.8309 9007062

[B44] McCullough K. C. De Simone F. Brocchi E. Capucci L. Crowther J. R. Kihm U. (1992). Protective immune response against foot-and-mouth disease. *J. Virol.* 66 1835–1840. 10.1128/jvi.66.4.1835-1840.1992 1312607 PMC288969

[B45] Medina G. N. Azzinaro P. Ramirez-Medina E. Gutkoska J. Fang Y. Diaz-San Segundo F. (2020). Impairment of the DeISGylation activity of foot-and-mouth disease virus lpro causes attenuation in vitro and in vivo. *J Virol.* 94:e341-20 10.1128/JVI.00341-20 PMC7307143 32295921

[B46] Minor P. D. (2015). Live attenuated vaccines: historical successes and current challenges. *Virology* 479-480 379–392. 10.1016/j.virol.2015.03.032 25864107

[B47] Moraes M. P. Chinsangaram J. Brum M. C. Grubman M. J. (2003). Immediate protection of swine from foot-and-mouth disease: a combination of adenoviruses expressing interferon alpha and a foot-and-mouth disease virus subunit vaccine. *Vaccine* 22 268–279. 10.1016/s0264-410x(03)00560-7 14615155

[B48] Moraes M. P. Segundo F. D. Dias C. C. Pena L. Grubman M. J. (2011). Increased efficacy of an adenovirus-vectored foot-and-mouth disease capsid subunit vaccine expressing nonstructural protein 2B is associated with a specific T cell response. *Vaccine* 29 9431–9440. 10.1016/j.vaccine.2011.10.037 22027486

[B49] Moss W. J. Strebel P. (2011). Biological feasibility of measles eradication. *J. Infect. Dis.* 204(Suppl. 1) S47–S53. 21666201 10.1093/infdis/jir065 PMC3112320

[B50] Moura G. Pinheiro M. Arrais J. Gomes A. C. Carreto L. Freitas A. (2007). Large scale comparative codon-pair context analysis unveils general rules that finetune evolution of mRNA primary structure. *PLoS One* 2:e847. 10.1371/journal.pone.0000847 17786218 PMC1952141

[B51] Mueller S. Coleman J. R. Papamichail D. Ward C. B. Nimnual A. Futcher B. (2010). Live attenuated influenza virus vaccines by computer aided rational design. *Nat. Biotechnol.* 28 723–726. 10.1038/nbt.1636 20543832 PMC2902615

[B52] Mueller S. Papamichail D. Coleman J. R. Skiena S. Wimmer E. (2006). Reduction of the rate of poliovirusprotein synthesis through large-scale codon deoptimization causes attenuation of viral virulence by lowering specific infectivity. *J. Virol.* 80 9687–9696. 10.1128/jvi.00738-06 16973573 PMC1617239

[B53] OIE (2018). *Manual of Diagnostic Tests and Vaccines for Terrestrial Animals. Part 2 OIE Listed Diseases and Other Diseases of Importance to International Trade. Section 2.1 Multiple Species. Chapter 3.1.8. Foot and Mouth Disease.* Paris: OIE, 433–464.

[B54] Park C. Baek J. H. Cho S. H. Jeong J. Chae C. You S. H. (2020). Field porcine reproductive and respiratory syndrome viruses (PRRSV) attenuated by codon pair deoptimization (CPD) in NSP1 protected pigs from heterologous challenge. *Virology* 540 172–183. 10.1016/j.virol.2019.10.019 31928999

[B55] Pay T. W. Hingley P. J. (1987). Correlation of 140S antigen dose with the serum neutralizing antibody response and the level of protection induced in cattle by foot-and-mouth disease vaccines. *Vaccine* 5 60–64. 10.1016/0264-410x(87)90011-9 3033928

[B56] Pluimers F. H. Akkerman A. M. van der Wal P. Dekker A. Bianchi A. (2002). Lessons from the foot and mouth disease outbreak in the Netherlands in 2001. *Rev. Sci. Tech.* 21 711–721. 10.20506/rst.21.3.1371 12523709

[B57] Rai D. K. Diaz-San Segundo F. Campagnola G. Keith A. Schafer E. A. Kloc A. (2017). Attenuation of foot-and-mouth disease virus by engineered viral polymerase fidelity. *J. Virol.* 91:e00081-17. 10.1128/JVI.00081-17 PMC5651715 28515297

[B58] Rieder E. Henry T. Duque H. Baxt B. (2005). Analysis of a foot-and-mouth disease virus type A24 isolate containing an SGD receptor recognition site in vitro and its pathogenesis in cattle. *J. Virol.* 79 12989–12998. 10.1128/jvi.79.20.12989-12998.2005 16189001 PMC1235811

[B59] Roeder P. Mariner J. Kock R. (2013). Rinderpest: the veterinary perspective on eradication. *Phil. Trans. R. Soc. B.* 368:20120139. 10.1098/rstb.2012.0139 23798687 PMC3720037

[B60] Salguero F. J. Sanchez-Martin M. A. Diaz-San Segundo F. de Avila A. Sevilla N. (2005). Foot-and-mouth disease virus (FMDV) causes an acute disease that can be lethal for adult laboratory mice. *Virology* 332 384–396. 10.1016/j.virol.2004.11.005 15661169

[B61] Shen S. H. Stauft C. B. Gorbatsevych O. Song Y. Ward C. B. Yurovsky A. (2015). Large-scale recoding of an arbovirus genome to rebalance its insect versus mammalian preference. *Proc. Natl. Acad. Sci. U.S.A.* 112 4749–4754. 10.1073/pnas.1502864112 25825721 PMC4403163

[B62] Simmonds P. (2012). SSE: a nucleotide and amino acid sequence analysis platform. *BMC Res Notes.* 5:50. 10.1186/1756-0500-5-50 22264264 PMC3292810

[B63] Stauft C. B. Shen S. H. Song Y. Gorbatsevych O. Asare E. Futcher B. (2018). Extensive recoding of dengue virus type 2 specifically reduces replication in primate cells without gain-of-function in Aedes aegypti mosquitoes. *PLoS One* 13:e0198303. 10.1371/journal.pone.0198303 30192757 PMC6128446

[B64] Stauft C. B. Song Y. Gorbatsevych O. Pantoja P. Rodriguez I. V. Futcher B. (2019a). Extensive genomic recoding by codon-pair deoptimization selective for mammals is a flexible tool to generate attenuated vaccine candidates for dengue virus 2. *Virology* 537 237–245. 10.1016/j.virol.2019.09.003 31539771

[B65] Stauft C. B. Yang C. Coleman J. R. Boltz D. Chin C. Kushnir A. (2019b). Live-attenuated H1N1 influenza vaccine candidate displays potent efficacy in mice and ferrets. *PLoS One* 14:e0223784. 10.1371/journal.pone.0223784 31609986 PMC6791556

[B66] Stenfeldt C. Arzt J. (2020). The carrier conundrum; a review of recent advances and persistent gaps regarding the carrier state of foot-and-mouth disease virus. *Pathogens* 9:167 10.3390/pathogens9030167 PMC7157498 32121072

[B67] Sutmoller P. McVicar J. W. Cottral G. E. (1968). The epizootiological importance of foot-and-mouth disease carriers. Experimentally produced foot-and-mouth disease carriers in susceptible and immune cattle. *Arch. Gesamte Virusforsch.* 23 227–235. 10.1007/bf01241895 5680590

[B68] Tulloch F. Atkinson N. J. Evans D. J. Ryan M. D. Simmonds P. (2014). RNA virus attenuation by codon pair deoptimisation is an artefact of increases in CpG/UpA dinucleotide frequencies. *Elife* 3 e04531. 10.7554/eLife.04531 PMC4383024 25490153

[B69] Uddowla S. Hollister J. Pacheco J. M. Rodriguez L. L. Rieder E. (2012). A safe foot-and-mouth disease vaccine platform with two negative markers for differentiating infected from vaccinated animals. *J. Virol.* 86 11675–11685. 10.1128/jvi.01254-12 22915802 PMC3486329

[B70] Uddowla S. Pacheco J. M. Larson C. Bishop E. Rodriguez L. L. Rai D. K. (2013). Characterization of a chimeric foot-and-mouth disease virus bearing a bovine rhinitis B virus leader proteinase. *Virology* 447 172–180. 10.1016/j.virol.2013.08.035 24210112

[B71] Velazquez-Salinas L. Risatti G. R. Holinka L. G. O’Donnell V. Carlson J. Alfano M. (2016). Recoding structural glycoprotein E2 in classical swine fever virus (CSFV) produces complete virus attenuation in swine and protects infected animals against disease. *Virology* 494 178–189. 10.1016/j.virol.2016.04.007 27110709

[B72] Visser L. J. Medina G. N. Rabouw H. H. de Groot R. J. Langereis M. A. de Los Santos T. (2019). Foot-and-mouth disease virus leader protease cleaves G3BP1 and G3BP2 and inhibits stress granule formation. *J. Virol.* 93: e00922-18. 10.1128/JVI.00922-18 PMC6321903 30404792

[B73] Wang D. Fang L. Li P. Sun L. Fan J. Zhang Q. (2011). The leader proteinase of foot-and-mouth disease virus negatively regulates the type I interferon pathway by acting as a viral deubiquitinase. *J. Virol.* 85 3758–3766. 10.1128/JVI.02589-10 21307201 PMC3126127

[B74] Wang B. Yang C. Tekes G. Mueller S. Paul A. Whelan S. P. (2015). Recoding of the vesicular stomatitis virus L gene by computer-aided design provides a live, attenuated vaccine candidate. *mBio* 6:e237-15. 10.1128/mBio.00237-15 PMC4453547 25827413

[B75] Xia X. (2020). Extreme genomic CpG deficiency in SARS-CoV-2 and evasion of host antiviral defense. *Mol. Biol. Evol.* 37 2699–2705. 10.1093/molbev/msaa094 32289821 PMC7184484

[B76] Yang C. Skiena S. Futcher B. Mueller S. Wimmer E. (2013). Deliberate reduction of hemagglutinin and neuraminidase expression of influenza virus leads to an ultraprotective live vaccine in mice. *Proc. Nat. Acad. Sci. U.S.A.* 110 9481–9486. 10.1073/pnas.1307473110 23690603 PMC3677463

[B77] Yeh M. T. Bujaki E. Dolan P. T. Smith M. Wahid R. Konz J. (2020). Engineering the Live-attenuated polio vaccine to prevent reversion to virulence. *Cell Host Microbe* 27 736–751. 10.1016/j.chom.2020.04.003 32330425 PMC7566161

[B78] Yu C. H. Dang Y. Zhou Z. Wu C. Zhao F. Sachs M. S. (2015). Codon usage influences the local rate of translation elongation to regulate co-translational protein folding. *Mol. Cell* 59 744–754. 10.1016/j.molcel.2015.07.018 26321254 PMC4561030

