# Supplementary figures and images for: Use of Synonymous Deoptimization to Derive Modified Live Attenuated Strains of Foot and Mouth Disease Virus
Source: Front Microbiol. 2021 Jan 21;11:610286. doi: 10.3389/fmicb.2020.610286 (PMC7861043; doi:10.3389/fmicb.2020.610286)

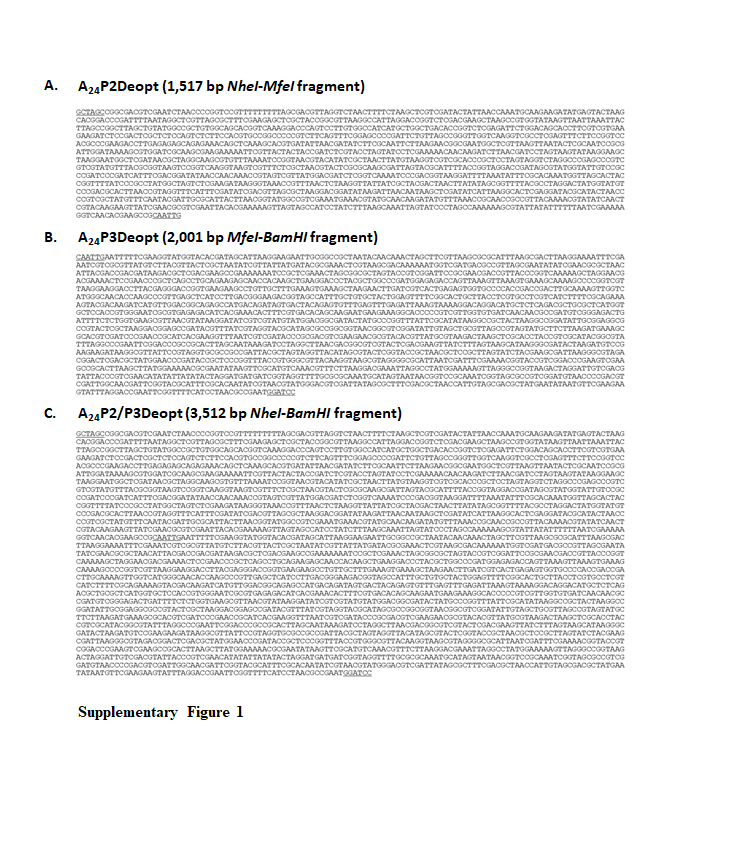

Supplement: Supplementary Figure 1 — Sequences of A24Cru with deoptimized (A) P2, (B) P3, and (C) P2/P3. Underlined are NheI, MfeI, and BamHI restriction sites. [file Image_1.tif]

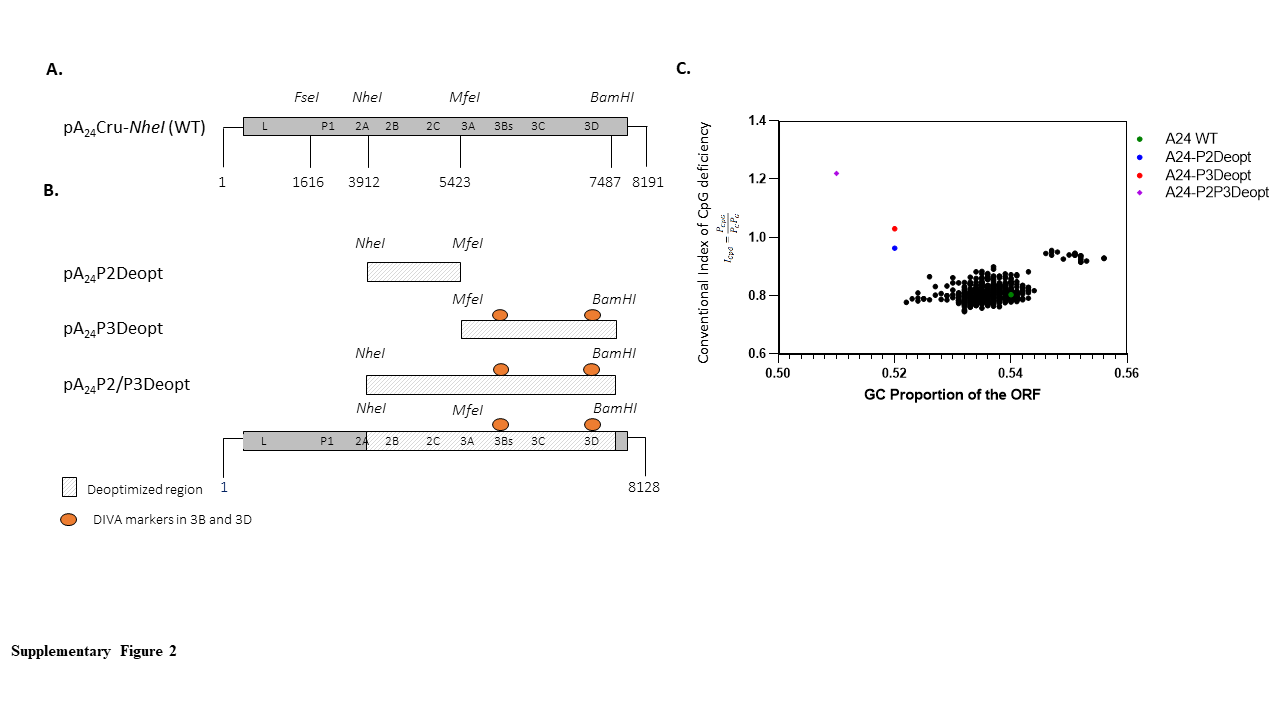

Supplement: Supplementary Figure 2 — Generation of deoptimized FMDV and sequence analysis. (A) Schematics of A24Cru wild type (WT) infectious clone with a unique added NheI site. Relevant restriction sites used for cloning are depicted (NheI, MfeI, and BamHI). (B) NheI/MfeI, MfeI/BamHI of NheI/BamHI fragments. containing deoptimized codons were synthesized and respectively replaced in pA24CruNheI. Synthesized fragments contained DIVA markers including a small deletion in 3B and amino acid substitutions in 3B and 3D. (C) Full polyprotein ORF from sequences obtained from National Center for Biotechnology Information (NCBI) website (http://www.ncbi.nlm.nih.gov) (n = 1055) were analyzed alongside the deoptimized strains in SSE V1.4 for nucleotide and dinucleotide frequencies (Simmonds, 2012). CpG odds ratio were calculated using the equation CpG odds ratio = fCpG/fCfG. [file Image_2.tif]
